# Tracking Cryptococcal Meningitis to Monitor HIV Program Success During the Treat All Era: An Analysis of National Data in Botswana

**DOI:** 10.1093/cid/ciae066

**Published:** 2024-02-08

**Authors:** James Milburn, Ookeditse Ntwayagae, Rachita Suresh, Kebatshabile Ngoni, Cassie Northcott, James Penney, Matthew Kinsella, Imogen Mechie, Samuel Ensor, Goitseone Thamae, Tshepo Leeme, David S Lawrence, Tony Chebani, Daniel Grint, Mark W Tenforde, Ava Avalos, Dinah Ramaabya, Justus Ogando, Margaret Mokomane, Madisa Mine, Joseph N Jarvis

**Affiliations:** Botswana Harvard Health Partnership, Gaborone, Botswana; Department of Clinical Research, Faculty of Infectious and Tropical Diseases, London School of Hygiene and Tropical Medicine, United Kingdom; Botswana–University of Maryland School of Medicine Health Initiative; Botswana Harvard Health Partnership, Gaborone, Botswana; Botswana Harvard Health Partnership, Gaborone, Botswana; Botswana Harvard Health Partnership, Gaborone, Botswana; Botswana Harvard Health Partnership, Gaborone, Botswana; Botswana Harvard Health Partnership, Gaborone, Botswana; Botswana Harvard Health Partnership, Gaborone, Botswana; Botswana Harvard Health Partnership, Gaborone, Botswana; Botswana Harvard Health Partnership, Gaborone, Botswana; Botswana Harvard Health Partnership, Gaborone, Botswana; Botswana Harvard Health Partnership, Gaborone, Botswana; Department of Clinical Research, Faculty of Infectious and Tropical Diseases, London School of Hygiene and Tropical Medicine, United Kingdom; Botswana Ministry of Health and Wellness, Gaborone; Department of Infectious Disease Epidemiology and International Health, Faculty of Epidemiology and Population Health, London School of Hygiene and Tropical Medicine, United Kingdom; Botswana–UPenn Partnership, Gaborone; Botswana Harvard Health Partnership, Gaborone, Botswana; Botswana Ministry of Health and Wellness, Gaborone; Clinton Health Access Initiative, Nairobi, Kenya; School of Allied Health Professions, University of Botswana, Gaborone, Botswana; National Health Laboratory, Ministry of Health and Wellness, Gaborone, Botswana; Botswana Harvard Health Partnership, Gaborone, Botswana; Department of Clinical Research, Faculty of Infectious and Tropical Diseases, London School of Hygiene and Tropical Medicine, United Kingdom

**Keywords:** cryptococcal meningitis, advanced HIV disease, opportunistic infections, Botswana

## Abstract

**Background:**

Cryptococcal meningitis (CM) causes substantial mortality in African countries with a high prevalence of human immunodeficiency virus (HIV), despite advances in disease management and increasing antiretroviral therapy (ART) coverage. Reliable diagnosis of CM is cheap and more accessible than other indicators of advanced HIV disease burden such as CD4 testing or investigation for disseminated tuberculosis; therefore, monitoring CM incidence has the potential to serve as a valuable metric of HIV programmatic success.

**Methods:**

Botswana national meningitis surveillance data from 2015 to 2022 were obtained from electronic health records. All electronic laboratory records from cerebrospinal fluid samples analyzed within government healthcare facilities in Botswana were extracted from a central online repository. Adjustments for missing data were made through triangulation with prospective cohort study datasets. CM case frequency was enumerated using a case definition and incidence calculated using national census data.

**Results:**

A total of 1744 episodes of CM were identified; incidence declined from 15.0 (95% confidence interval [CI], 13.4–16.7) cases/100 000 person-years in 2015 to 7.4 (95% CI, 6.4–8.6) cases/100 000 person-years in 2022. However, the rate of decline slowed following the introduction of universal treatment in 2016. The highest incidence was observed in men and individuals aged 40–44 years. The proportion of cases diagnosed through cryptococcal antigen testing increased from 35.5% to 86.3%.

**Conclusions:**

CM incidence has decreased in Botswana following expansion of ART coverage but persists at a stubbornly high incidence. Most cases are now diagnosed through the cheap and easy-to-use cryptococcal antigen test, highlighting the potential of using CM as key metric of program success in the Treat All era.

## Teaser

Cryptococcal meningitis remains a leading cause of mortality in high-HIV-prevalence settings. In Botswana, cryptococcal meningitis incidence declined with expanded antiretroviral coverage. Rapid cryptococcal antigen test was increasingly used, highlighting cryptococcal meningitis surveillance as an important metric of HIV programmatic success.

Cryptococcal meningitis remains the most common cause of meningitis in sub-Saharan Africa, typically affecting patients with advanced human immunodeficiency virus (HIV) disease (AHD) [1]. Despite widespread expansion of antiretroviral therapy (ART) programs, modeled estimates suggest that although there has been some reduction in global incidence of cryptococcal meningitis, it remains a major cause of mortality among people with HIV (PWH), accounting for 19% of all HIV-related deaths globally [1]. However, very few countries collect reliable statistics on cryptococcal meningitis incidence. Therefore, the impact of World Health Organization (WHO) universal HIV treatment (Treat All) guidelines introduced in 2016 and subsequent expansions in ART coverage on the incidence of opportunistic infections (OIs) such as cryptococcal meningitis is not known.

Botswana has been at the forefront of ART programming in Africa; it was the first African country to offer free ART to citizens in 2002 at a time when the national HIV prevalence was >25% [2], and a series of innovative HIV care models were implemented including the adoption of universal treatment in 2016 [3]. Under the Treat All strategy, any person who tested positive for HIV should be started on treatment, regardless of CD4 count or viral load, and in 2022 Botswana became one of the first countries globally to report reaching the Joint United Nations Programme on HIV/AIDS (UNAIDS) 95-95-95 targets. In the context of such extensive ART coverage, the incidence of AHD and the rates of OIs such as cryptococcal meningitis would be expected to decline. However, adult HIV prevalence rates remain high at 18.6% in those aged 15–49 years in 2021 [4], and presentations with AHD remain common through late diagnosis or disengagement with treatment [5–8]. Previous data from Botswana demonstrated that cryptococcal meningitis incidence initially fell following widespread ART rollout in the mid-2000s but plateaued between 2010 and 2014 [9], with the 2013–2014 cryptococcal meningitis incidence in Botswana comparable to pre-ART rates in neighboring South Africa. More recent data from South Africa demonstrated that the incidence of cryptococcal disease varies between regions, with some districts reporting an increase in incidence from 2018 to 2019 [10], highlighting that even in countries with high levels of ART coverage there remains a significant population of individuals developing AHD and associated OIs.

Cryptococcal meningitis is an important and potentially accessible metric to assess the performance of national HIV program success, although to date it has not been widely utilized due to the lack of established data collection systems. The majority of individuals who develop cryptococcal meningitis will present to healthcare facilities, and the disease can be easily and reliably diagnosed using cheap, highly sensitive, and easy-to-use cryptococcal antigen (CrAg) lateral flow assays. The IMMY CrAg lateral flow assay (IMMY, Norman, Oklahoma) is widely used in Botswana and can be performed in <15 minutes without significant laboratory infrastructure or training [11]. This is in marked contrast to many other indicators of AHD, such as CD4 count testing, which requires extensive laboratory infrastructure [12], or other indicator diseases such as disseminated tuberculosis or pneumocystis pneumonia (PCP), where there is a lack of sensitive diagnostics, often considerable diagnostic uncertainty clinically, and a large proportion of disease in the community rather than healthcare facilities, making accurate case ascertainment difficult [13, 14].

To explore the utility of cryptococcal meningitis surveillance in assessing the impact of national HIV programs, and to establish the impact of the Treat All strategy introduction in 2016 on cryptococcal meningitis incidence in the high-HIV-prevalence setting of Botswana, we analyzed 8 years of routine national laboratory data from the Botswana Ministry of Health and Wellness electronic medical record systems alongside data regarding ART coverage from the National ART Programme.

## METHODS

### Study Design

The Botswana National Meningitis Survey is an ongoing meningitis surveillance network utilizing routine national data to monitor trends in the etiology of central nervous system infections in Botswana [15]. Periodic review of national electronic laboratory records of cerebrospinal fluid samples (CSF) collected between 1 January 2015 and 31 December 2022 was undertaken. Between 2015 and 2022, CSF analysis was performed at government laboratories linked to 25 healthcare facilities: 2 referral hospitals, 7 district hospitals, and 16 primary hospitals. Universal healthcare, including CSF analysis and CrAg testing, is provided for free to Botswana citizens. Routine analysis of CSF samples in Botswana should consist of macroscopic examination and cell count with differential if white cell count is ≥10 cells/μL. The sample is centrifuged and Gram and India ink stained, and culture is performed on the sediment using Sabouraud dextrose, blood agar, and chocolate agar. CrAg testing is performed on uncentrifuged CSF samples from all adult patients ≥18 years of age and upon request for pediatric cases. However, these tests are reliant on receipt of sufficient volume of CSF and adequate supply of consumables. Therefore not all tests are performed on all samples. Laboratory records from laboratories performing CSF analysis are uploaded on to a national electronic health record system, the Integrated Patient Management System (IPMS). All CSF samples with results stored on IPMS were extracted from a centralized online repository in collaboration with the Botswana Ministry of Health and Wellness. There are 3 private hospitals that do not report to IPMS and therefore data from these hospitals were not captured. In 2014, based on a comprehensive nationwide surveillance study, the private sector accounted for 7.4% of all samples [16]. We have assumed that the proportional public/private workload has remained constant and applied this figure as an adjustment to our estimates. One hospital linked to a mining development did not report results to IPMS. This hospital had between 11 and 14 cases of cryptococcal meningitis annually from the same nationwide data; we applied an additional 2.5% uplift to incidence estimates to account for this.

Data capture in the electronic IPMS system is not 100% complete due to intermittent power outages, as well as poor internet connectivity or maintenance (known as “downtime”). In periods of IPMS downtime, results are disseminated locally on paper records. To correct for this incomplete coverage, data from the national referral hospital, Princess Marina Hospital (PMH), Gaborone, were used to triangulate the underestimation of cryptococcal meningitis cases due to periods of IPMS downtime. Comprehensive prospective data including all paper downtime records were collected from every patient with CSF submitted to PMH in 2022, which accounts for approximately 40% of all CSF samples analyzed in Botswana. In this prospective cohort, 34 cases of cryptococcal meningitis were diagnosed, 31 of which were identified on IPMS, an underestimation of 8.8%. As PMH is the national referral hospital in Botswana and has more robust information technology infrastructure than smaller regional or district hospitals where there will be a larger amount of CSF results reported on paper not captured in this study, cryptococcal meningitis case frequency and incidence rates were inflated by a conservative 10% to account for this underestimation.

### Cryptococcal Meningitis Case Definition

A case of cryptococcal meningitis was defined as a positive CSF India ink stain, positive CSF culture for *Cryptococcus neoformans*, and/or positive CSF CrAg. As CSF analysis can be repeated on patients with cryptococcal meningitis for management of raised intracranial pressure, when >1 CSF sample was analyzed for an individual patient, an episode was defined as a positive CSF sample >14 days apart from a previous positive sample. Positive samples ≤14 days from each other were considered part of the same episode.

### Data Analysis

The number of cases were enumerated using the case definition. Patient age and sex were described using frequencies, percentages, or median and interquartile range (IQR) as appropriate. 2011 and 2021 census data were used to calculate cryptococcal meningitis incidence. A linear increase in population across intervening years was assumed to determine yearly cryptococcal meningitis incidence. Breakdown of population by sex is currently not available for the 2022 census; therefore, the same proportion of males and females from the 2011 census was assumed across all years. UNAIDS Spectrum model data were used to determine HIV prevalence and number of individuals receiving ART. The 95% confidence intervals (CIs) for incidence of cryptococcal meningitis were derived using the exact binomial method. Linear regression analysis was performed to assess the relationship between ART coverage among PWH and cryptococcal meningitis incidence.

The frequency of cryptococcal meningitis case diagnosis at the 2 national referral hospitals (PMH and Nyangabgwe Referral Hospital, Francistown) was compared before and after the introduction of Treat All in June 2016 using data shared from the previous national analysis [9]. This longitudinal analysis was restricted to the 2 referral hospitals as these were the only sites with comprehensive longitudinal data pre-2015.

Interrupted time series analysis was performed to assess the effect of Treat All as an intervention to decrease cryptococcal meningitis case frequency. Yearly cryptococcal meningitis case frequency data were smoothed using a moving average and Newey-West standard errors for coefficients were estimated by ordinary least squares regression. The intervention cut-point was set at 1 January 2017 to allow a lead-in time of 6 months for patients to be established on ART following the change in the national ART program strategy.

### Ethical Approvals

The study was performed with the support of the Botswana Ministry of Health and Wellness. Institutional review board approval was in place from the Health Research Development Committee (Botswana Ministry of Health and Wellness), London School of Hygiene and Tropical Medicine, and University of Botswana, and local approval was obtained from the study's sentinel site (PMH).

## RESULTS

A total of 1744 episodes of cryptococcal meningitis were identified occurring in 1440 individuals between 2015 and 2022 (Table 1). In patients diagnosed with cryptococcal meningitis, 84.5% (1217/1440) had a single episode of cryptococcal meningitis and 15.4% (223/1440) had 2 or more episodes. The median time between first and second episode was 48 days (IQR, 19–130 days). The median age at diagnosis was 38.9 years (IQR, 33.1–45.5 years) with more cases in males (938/1440 [65.1%]) than females; 44.2% (770/1744) of all cryptococcal meningitis cases were diagnosed in 1 of the 2 national referral hospitals and 33.2% (579/1744) were diagnosed in district hospitals. The remainder were diagnosed either in primary hospitals, clinics, or hospital linked to mining developments.

**Table 1.** Description of Basic Demographics and CD4 Count of Patients With Cryptococcal Meningitis and Cryptococcal Meningitis Diagnoses in Botswana by Month

Variable	No. (%) or Median (IQR)
Age at diagnosis^a^ (n = 1439/1440)	
Median (IQR)	38.9 (33.1–45.5)
Sex^a^ (n = 1440/1440)	
Male	938 (65.1)
Female	502 (34.9)
CD4 count at diagnosis, cells/μL (closest result within 6 mo of LP)^b^ (n = 944/1744)	
Median (IQR)	48 (23–107)
Month of diagnosis^b^ (n = 1744/1744)	
January	143 (8.2)
February	126 (7.2)
March	164 (9.4)
April	157 (9.0)
May	149 (8.5)
June	125 (7.2)
July	152 (8.7)
August	156 (8.9)
September	136 (7.8)
October	169 (9.7)
November	149 (8.5)
December	118 (6.8)

For CD4 testing the closest result to the date of cryptococcal meningitis diagnosis was reported if it was within a 6-month window of the diagnosis of cryptococcal meningitis.

Abbreviations: IQR, interquartile range; LP, lumbar puncture.

^a^De-duplicated to represent individual patients rather than episodes.

^b^Data from all episodes including relapses.

**Figure 1.**
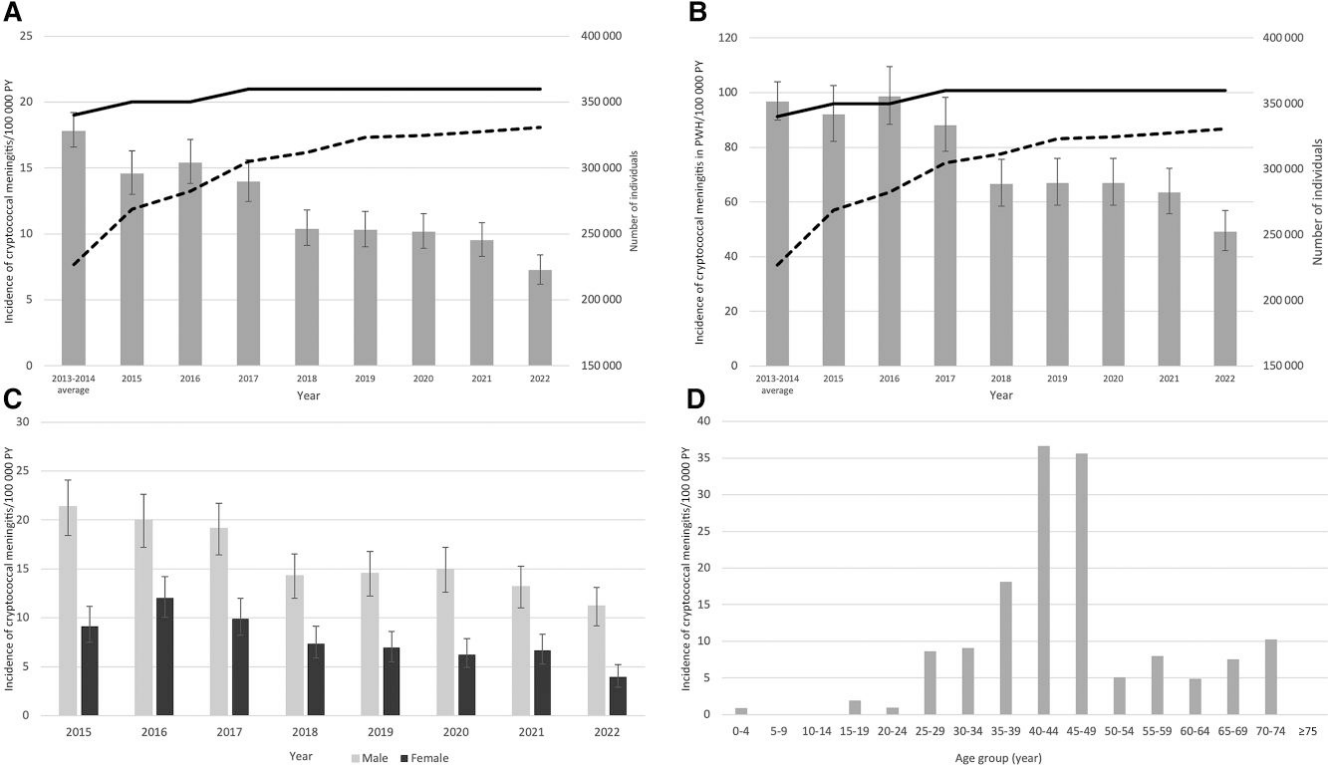
*A*, Incidence of cryptococcal meningitis in Botswana/100 000 person-years (PY) of observation from 2013 to 2022 (bar chart) with 95% confidence intervals (CIs). Joint United Nations Programme on HIV/AIDS (UNAIDS) estimate of total numbers of people with human immunodeficiency virus (PWH) (unbroken line) and number of people receiving antiretroviral therapy (dotted line). *B*, Incidence of cryptococcal meningitis/100 000 PY of observation in PWH between 2013 and 2022 with 95% CIs. UNAIDS estimate of total numbers of PWH (unbroken black line) and number of people receiving antiretroviral therapy (dotted line). *C*, Incidence of cryptococcal meningitis/100 000 PY of observation by sex between 2015 and 2022 with 95% CIs. *D*, Incidence of cryptococcal meningitis/100 000 PY of observation by age category in 2022.

The estimated national incidence of cryptococcal meningitis in Botswana approximately halved between 2015 and 2022 (15.0 [95% CI, 13.4–16.7] cases/100 000 person-years and 7.4 [95% CI, 6.4–8.6] cases/100 000 person-years, respectively) (Figure 1*A*). In PWH, the incidence of cryptococcal meningitis decreased from 92.0 (95% CI, 82.2–102.6) cases/100 000 person-years in 2015 to 49.1 (95% CI, 42.2–57.0) cases/100 000 person-years in 2022 (Figure 1*B*). The incidence of cryptococcal meningitis decreased in both males and females between 2015 and 2022 (Figure 1*C*). Incidence remained nearly 3-fold higher in males in 2022, with 11.2 cases/100 000 person-years in males and 4.0 cases/100 000 person-years in females. Peak incidence by age was between 40 and 44 years (Figure 1*D*). Linear regression analysis of the association between cryptococcal meningitis incidence and proportion of PWH on ART showed that for every 5% increase in ART coverage, we observed a decrease in cryptococcal meningitis incidence of 2.5 cases/100 000 person-years.

**Figure 2.**
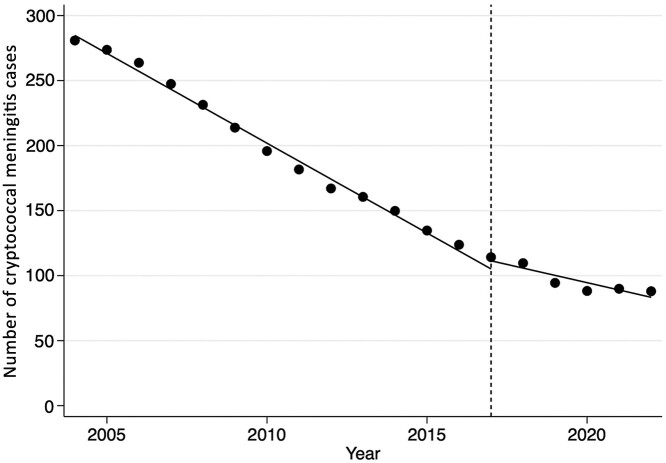
Frequency of cryptococcal meningitis cases at the 2 national referral hospitals between 2004 and 2023. Interrupted time series analysis was used to generate predicted trends in cryptococcal meningitis cases pre- and postintervention.

The frequency of cryptococcal meningitis cases at the 2 national referral hospitals decreased between 2004 and 2022 (Figure 2). There was no significant decline in the case frequency of cryptococcal meningitis at the point of Treat All introduction, with a change in case frequency of 9.5 (95% CI, −1.4 to 20.3; *P* = .082) observed. The rate of decline in cryptococcal meningitis cases/year before the intervention date of 1 January 2017 was −13.0 cases/year (95% CI, −13.9 to −12.1; *P* < .001). The rate of decline after the intervention date was −6.2 cases/year (95% CI, −8.5 to −3.9; *P* < .001). The test for interaction between intervention period and time provided strong evidence that the rate of decline after the intervention date was lower than before (*P* < .001).

**Figure 3.**
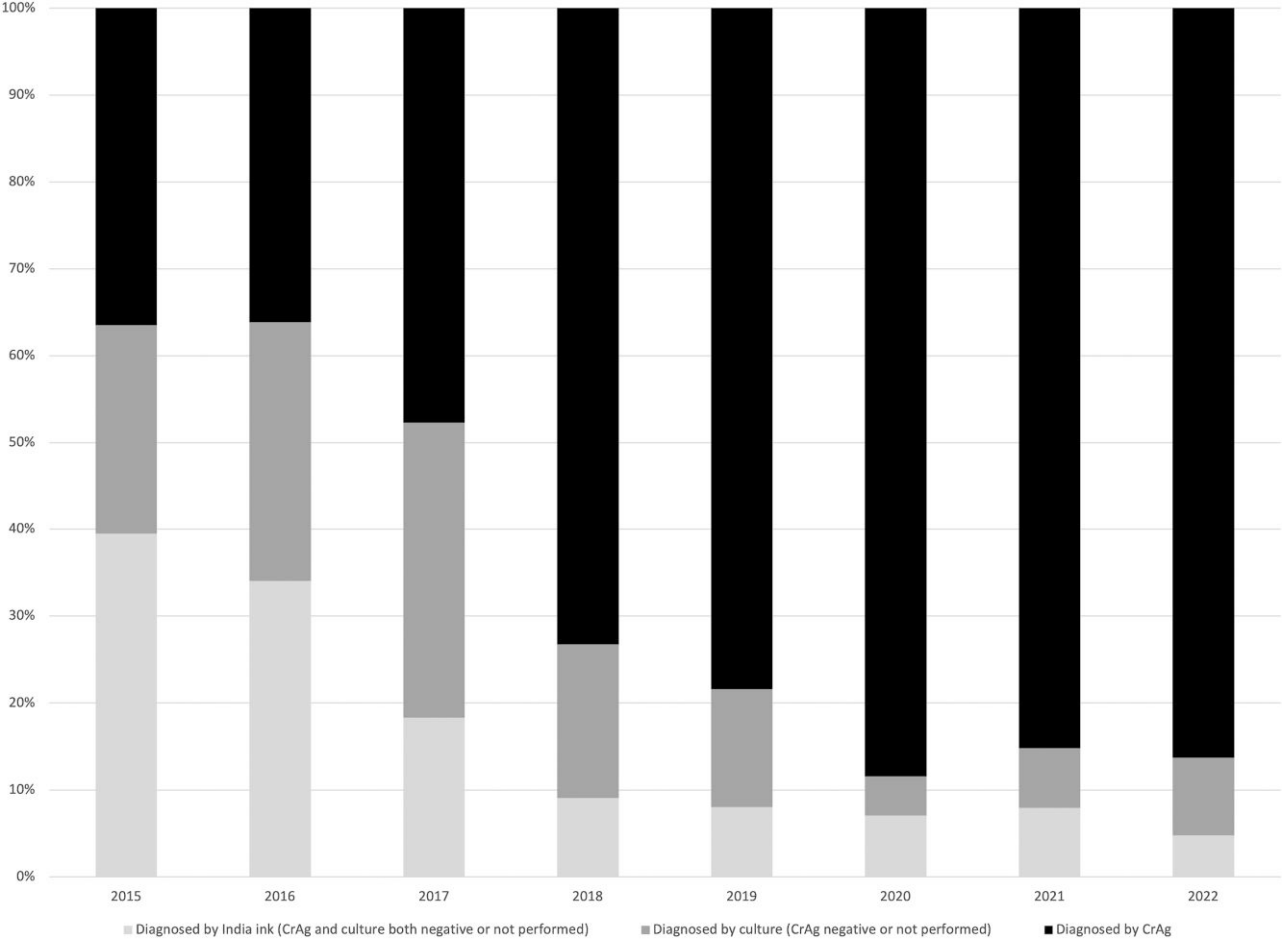
Yearly variation in cryptococcal meningitis diagnoses made through different diagnostic modalities. Group definitions are as follows: Diagnosed by cryptococcal antigen (CrAg): positive cerebrospinal fluid (CSF) CrAg; India ink testing and culture positivity not accounted for. Diagnosed by culture: positive CSF culture for *Cryptococcus neoformans*. CSF CrAg testing either negative or not performed; India ink testing not accounted for. Diagnosed by India ink: positive CSF India ink stain. CSF CrAg and culture both negative or not performed.

The proportions of cryptococcal meningitis diagnoses made through the 3 most common diagnostic modalities (India ink, culture, and CrAg testing) available in Botswana are displayed in Figure 3. CrAg testing became the most used modality, with the proportion of diagnoses made through CrAg testing increasing from 35.5% in 2015 to 86.3% in 2022.

## DISCUSSION

Robust national cryptococcal meningitis incidence estimates from Botswana, an African country with high HIV prevalence, demonstrate that cryptococcal meningitis incidence has declined since 2015 with the narrowing ART treatment gap, but this rate of decline in cryptococcal meningitis incidence has slowed despite increasing ART coverage following the rollout of universal treatment in 2016. While individual patient–level data are lacking to make direct causal observations, our data demonstrated that the incidence of cryptococcal meningitis was significantly associated with the proportion of patients on ART. Patients presenting with cryptococcal meningitis in Botswana typically had a CD4 count <50 cells/μL, highlighting that a hard-to-reach population, with an overrepresentation of working age males, remains at risk of cryptococcal meningitis usually due to late diagnosis or cycling out of treatment services due to the failure of services to effectively engage and retain this group. Providing effective care for this group will require novel treatment strategies and enhanced OI screening and prevention to reduce presentations with AHD and associated OIs.

While the incidence of cryptococcal meningitis continued to decline after 2017, the rate of decline slowed compared to the period before 2017. In contrast, we might have expected to see the ongoing impact of universal HIV treatment to increase the rate of decline in cryptococcal meningitis incidence during this period. It is likely that during that period the coronavirus disease 2019 (COVID-19) pandemic adversely affected HIV care, as demonstrated in other countries where service utilization was decreased in a number of diseases [17, 18]. Botswana had one of the highest reported mortality rates from COVID-19 in Africa, and access to healthcare including HIV services was challenging due to COVID-19–related restrictions [19]. As such, it is possible that interruptions to HIV care during the peak of the COVID-19 pandemic prevented further decline in cryptococcal meningitis incidence, and continued monitoring is necessary to establish the true impact of universal treatment.

We have demonstrated the potential for cryptococcal meningitis to be a key metric for monitoring HIV program success in the Treat All era using national laboratory data from a high-HIV-prevalence country. Botswana is uniquely placed in the region to conduct nationwide surveillance of AHD presentations with cryptococcal meningitis due to a robust electronic health record system where patients are linked through a national identification number. These data show that continued presentations with AHD in Botswana are common even with widespread ART coverage. Patients presenting with AHD have increased mortality compared to those who do not present with AHD, and monitoring and addressing excess mortality from AHD is crucial to inform program success [20, 21]. However, capturing these data is often challenging. Accurate mortality data are lacking as very few high-HIV-prevalence countries have reliable systems in place to track mortality or cause of death. Furthermore, AHD is often not identified, as a reliance on WHO clinical staging alone misses a high proportion of patients with AHD [22], and since the advent of Treat All, routine preinitiation CD4 count testing has not been prioritized. CD4 testing requires significant laboratory infrastructure and regular supply of consumables, which, since donor funding for CD4 testing has been cut, are often not available. Therefore, other indicators of AHD are necessary. Screening for OIs such as disseminated tuberculosis or PCP is difficult as diagnoses cannot be made confidently on clinical findings alone and available diagnostics are often costly or insensitive. Cryptococcal meningitis can be diagnosed using the affordable, highly sensitive CrAg test and in contrast to other OIs, most cases of cryptococcal meningitis will be seen in healthcare facilities, making case number ascertainment easier. Our data confirm that the majority of cryptococcal meningitis cases are now diagnosed through CrAg testing, making cryptococcal meningitis a cheap and accessible metric for AHD surveillance and valuable indicator HIV program success.

There are some important limitations to the study. We derived incidence estimates solely from data stored on electronic health records, and not all results will be uploaded to this system due to interruptions in connection to the database or the testing facility reporting results to clinicians through a different modality. Although we used existing, reliable datasets to triangulate for this underestimation and account for this using conservative percentage uplifts in our estimates, this undercounting may not have been fully corrected for, and our corrections add an additional degree of uncertainty to our estimates. Some individuals will not seek medical care or die before being diagnosed with cryptococcal meningitis. Further underestimation may occur as some facilities are unable to reliably diagnosis cryptococcal meningitis due to a lack of clinical equipment such as lumbar puncture needles or laboratory reagents, including CrAg kits, to test for cryptococcal meningitis. As such, the estimates presented here are likely at the lowest range of cryptococcal meningitis incidence. ART coverage estimates were derived from UNAIDS Spectrum model data and we were unable to confirm the accuracy of these data as we did not capture ART status data. Cryptococcal meningitis case numbers were enumerated using a 2-week interval between lumbar punctures. While this cutoff has been used previously, CSF CrAg positivity can persist for >2 weeks, so in a small number of cases there may be some ambiguity as to whether a positive CSF CrAg represents a new infection. CSF CrAg testing is more sensitive than culture and India ink; the increased utilization of CSF CrAg to diagnose cryptococcal meningitis during the study period may have resulted in the detection of cases that would have otherwise been missed through India ink and culture alone. Therefore, lower estimates of cryptococcal meningitis incidence may have been observed in those years when the majority of cryptococcal meningitis cases were diagnosed through India ink or culture. While there was an association between ART coverage and cryptococcal meningitis incidence, the impact of other interventions such as CrAg screening programs have not been accounted for. CrAg screening was introduced in Botswana in 2016, but initially limited to small pilot programs in the capital city, with wider rollout not occurring until 2019; given that our data are restricted to CSF testing results, we were unable to account for the impact of blood CrAg screening programs, which may have also contributed to a decline in cryptococcal meningitis incidence, particularly in the last 2–3 years of observation. Analyses to determine the reach and impact of CrAg screening programs in Botswana are in progress.

Monitoring and understanding of mortality from AHD forms an important part of HIV programming, but mortality data are often lacking and many indicators of AHD are either inaccessible due to cost or infrastructure, insensitive, or used on predominantly outpatient populations, making data collection more difficult. Cryptococcal meningitis incidence persists, disproportionately affecting key populations despite excellent ART coverage. Cryptococcal meningitis surveillance is therefore a potentially reliable and accessible metric that could be expanded to be a key monitoring marker to evaluate HIV program success.

## Notes

***Author Contributions*.** J. N. J., M. T. and J. M. conceived the project. J. M., O. N., R. S., K. N., C. N., J. P., M. K., I. M., S. E., A. A., D. R. and T. C. were involved in data extraction and data generation. J. M. performed the analysis with guidance from D. G. and J. N. J. All authors contributed to the manuscript writing, reviewing and approval of the final version.

***Disclaimer.*** The views expressed in this publication are those of the author(s) and not necessarily those of the National Institute for Health and Care Research or the UK Department of Health and Social Care.

***Financial support*.** This work was supported by the National Institute for Health and Care Research (NIHR) through a Global Health Research Professorship to J. N. J. (RP-2017-08-ST2- 012) using UK aid from the UK government to support global health research.

## References

[1] Rajasingham R , Govender NP, Jordan A, et al The global burden of HIV-associated cryptococcal infection in adults in 2020: a modelling analysis. Lancet Infect Dis 2022; 22:1748–55. 36049486 10.1016/S1473-3099(22)00499-6 PMC9701154

[2] Stover J , Fidzani B, Molomo BC, Moeti T, Musuka G. Estimated HIV trends and program effects in Botswana. PLoS One 2008; 3:e3729. 19008957 10.1371/journal.pone.0003729 PMC2579326

[3] Vinikoor MJ , Hachaambwa L. Advanced HIV disease during the ‘Treat All’ era in Botswana. AIDS 2020; 34:2321–3. 33196496 10.1097/QAD.0000000000002701 PMC8137811

[4] Joint United Nations Programme on HIV/AIDS (UNAIDS) . HIV and AIDS estimates Botswana. 2022. Available at: https://www.unaids.org/en/regionscountries/countries/botswana. Accessed 26 June 2023.

[5] Jarvis JN , Lawrence DS, Meya DB, et al Single-dose liposomal amphotericin B treatment for cryptococcal meningitis. N Engl J Med 2022; 386:1109–20. 35320642 10.1056/NEJMoa2111904 PMC7612678

[6] Lebelonyane R , Mills LA, Mogorosi C, et al Advanced HIV disease in the Botswana combination prevention project: prevalence, risk factors, and outcomes. AIDS 2020; 34:2223–30. 32694412 10.1097/QAD.0000000000002627

[7] Leeme TB , Mine M, Lechiile K, et al Utility of CD4 count testing in the era of universal ART: an analysis of routine laboratory data in Botswana. HIV Med 2021; 22:1–10. 10.1111/hiv.12951 PMC7736557 32876378

[8] Tenforde MW , Milton T, Rulaganyang I, et al Outcomes of reflex cryptococcal antigen (CrAg) screening in human immunodeficiency virus (HIV)-positive patients with CD4 counts of 100–200 cells/μL in Botswana. Clin Infect Dis 2021; 72:1635–8. 32604411 10.1093/cid/ciaa899 PMC8096249

[9] Tenforde MW , Mokomane M, Leeme T, et al Advanced human immunodeficiency virus disease in Botswana following successful antiretroviral therapy rollout: incidence of and temporal trends in cryptococcal meningitis. Clin Infect Dis 2017; 65:779–86. 28505328 10.1093/cid/cix430 PMC5850554

[10] Desanto DJ , Bangdiwala AS, Van Schalkwyk E, et al Evaluation of the effectiveness of a South African laboratory cryptococcal antigen screening programme using a retrospective cohort and a cluster-randomised trial design. BMJ Open 2022; 12:e054057.

[11] Huang HR , Fan LC, Rajbanshi B, Xu JF. Evaluation of a new cryptococcal antigen lateral flow immunoassay in serum, cerebrospinal fluid and urine for the diagnosis of cryptococcosis: a meta-analysis and systematic review. PLoS One 2015; 10:e0127117. 25974018 10.1371/journal.pone.0127117 PMC4431798

[12] Pham MD , Agius PA, Romero L, et al Acceptability and feasibility of point-of-care CD4 testing on HIV continuum of care in low and middle income countries: a systematic review. BMC Health Serv Res 2016; 16:343. 27484023 10.1186/s12913-016-1588-y PMC4971709

[13] Bateman M , Oladele R, Kolls JK. Diagnosing *Pneumocystis jirovecii* pneumonia: a review of current methods and novel approaches. Med Mycol 2020; 58:1015–28. 32400869 10.1093/mmy/myaa024 PMC7657095

[14] Sharma SK , Mohan A, Kohli M. Extrapulmonary tuberculosis. Expert Rev Respir Med 2021; 15:931–48. 33966561 10.1080/17476348.2021.1927718

[15] Tenforde MW , Mokomane M, Leeme T, et al Epidemiology of adult meningitis during antiretroviral therapy scale-up in southern Africa: results from the Botswana national meningitis survey. J Infect 2019; 79:212–9. 31255634 10.1016/j.jinf.2019.06.013 PMC6679721

[16] Tenforde MW , Mokomane M, Leeme TB, et al Mortality in adult patients with culture-positive and culture-negative meningitis in the Botswana national meningitis survey: a prevalent cohort study. Lancet Infect Dis 2019; 19:740–9. 31250824 10.1016/S1473-3099(19)30066-0 PMC7645732

[17] Soko RN , Burke RM, Feasey HRA, et al Effects of coronavirus disease pandemic on tuberculosis notifications, Malawi. Emerg Infect Dis 2021; 27:1831–9. 34152962 10.3201/eid2707.210557 PMC8237899

[18] McFall AM , Menezes NP, Srikrishnan AK, et al Impact of the COVID-19 pandemic on HIV prevention and care services among key populations across 15 cities in India: a longitudinal assessment of clinic-based data. J Int AIDS Soc 2022; 25:e25960. 35818314 10.1002/jia2.25960 PMC9273869

[19] Ensor S , Mechie I, Ryan R, et al Measuring the impact of COVID-19 social distancing measures on sexual health behaviours and access to HIV and sexual and reproductive health services for people living with HIV in Botswana. Front Global Womens Health 2023; 4:981478. 10.3389/fgwh.2023.981478 PMC10030995 36970120

[20] Walker AS , Prendergast AJ, Mugyenyi P, et al Mortality in the year following antiretroviral therapy initiation in HIV-infected adults and children in Uganda and Zimbabwe. Clin Infect Dis 2012; 55:1707–18. 22972859 10.1093/cid/cis797 PMC3501336

[21] Boyd AT , Oboho I, Paulin H, et al Addressing advanced HIV disease and mortality in global HIV programming. AIDS Res Ther 2020; 17:40. 32650797 10.1186/s12981-020-00296-x PMC7348123

[22] Munthali C , Taegtmeyer M, Garner PG, et al Diagnostic accuracy of the WHO clinical staging system for defining eligibility for ART in sub-Saharan Africa: a systematic review and meta-analysis. J Int AIDS Soc 2014; 17:18932. 24929097 10.7448/IAS.17.1.18932 PMC4057784

